# Molecular phylogeny of *Anopheles hyrcanus* group (Diptera: Culicidae) based on mtDNA *COI*

**DOI:** 10.1186/s40249-017-0273-7

**Published:** 2017-05-08

**Authors:** Yuan Fang, Wen-Qi Shi, Yi Zhang

**Affiliations:** National Institute of Parasitic Diseases, Chinese Center for Disease Control and Prevention; WHO Collaborating Centre for Tropical Diseases; National Center for International Research on Tropical Diseases, Ministry of Science and Technology; Key Laboratory of Parasite and Vector Biology, Ministry of Health, Shanghai, 200025 China

**Keywords:** Mosquito, DNA barcoding, Genetic distance, Malaria

## Abstract

**Background:**

The *Anopheles hyrcanus* group, which includes at least 25 species, is widely distributed in the Oriental and Palearctic regions. Some group members have been incriminated as vectors of malaria and other mosquito-borne diseases. It is difficult to identify Hyrcanus Group members by morphological features. Thus, molecular phylogeny has been proposed as an important complementary method to traditional morphological taxonomy.

**Methods:**

Based on the GenBank database and our original study data, we used 466 mitochondrial DNA *COI* sequences belonging to 18 species to reconstruct the molecular phylogeny of the Hyrcanus Group across its worldwide geographic range.

**Results:**

The results are as follows. 1) The average conspecific K2P divergence was 0.008 (range 0.002–0.017), whereas sequence divergence between congroup species averaged 0.064 (range 0.026–0.108). 2) The topology of *COI* tree of the Hyrcanus Group was generally consistent with classical morphological taxonomy in terms of species classification, but disagreed in subgroup division. In the *COI* tree, the group was divided into at least three main clusters. The first cluster contained *An. nimpe*; the second was composed of the Nigerrimus Subgroup and *An. argyropus*; and the third cluster was comprised of the Lesteri Subgroup and other unassociated species. 3) Phylogenetic analysis of *COI* indicated that ancient hybridizations probably occurred among the three closely related species, *An. sinensis*, *An. belenrae*, and *An. kleini*. 4) The results supported *An. paraliae* as a probable synonym of *An. lesteri*, and it was possible that *An. pseudopictus* and *An. hyrcanus* were the same species, as evident from their extremely low interspecific genetic divergence (0.020 and 0.007, respectively) and their phylogenetic positions.

**Conclusions:**

In summary, we reconstructed the molecular phylogeny and analysed genetic divergence of the Hyrcanus Group using mitochondrial *COI* sequences. Our results suggest that in the future of malaria surveillance, we should not only pay much attention to those known vectors of malaria, but also their closely related species.

**Electronic supplementary material:**

The online version of this article (doi:10.1186/s40249-017-0273-7) contains supplementary material, which is available to authorized users.

## Multilingual abstracts

Please see Additional file 1 for translations of the abstract into the five official working languages of the United Nations.

## Backgrounds

According to the *Action Plan of China Malaria Elimination* (APCME), *2010*–*2020*, most areas in China should have been malaria-free since 2015, except for the Yunnan Province. To solidify achievements and realize the goal of nationwide malaria elimination by 2020, the government must address concerns regarding the *Plasmodium* reservoirs, including surveillance of the remaining parasite reservoirs, and monitoring insecticide resistance in vector populations [1]. The primary malaria vectors in China are *Anopheles sinensis* Wiedemann, 1828, *An. lesteri* Baisas & Hu, 1936, *An. dirus* Peyton & Harrison, 1979, and *An. minimus* (Theobald, 1905) [2]. The distribution ranges of the former two are mainly in the elimination areas, whereas the latter two are a major threat in Yunnan Province [3]. All of them belong to the genus *Anopheles. An. sinensis* and *An. lesteri* are species in the Hyrcanus Group [4]. There are 25 recognized species in this group according to Harbach [4], and one provisionally designated member, *An. hyrcanus* sp_IR_ [5]. The group comprises several species that transmit not only malaria [6–9] and filariasis [10, 11], but also Japanese encephalitis virus [12–14]. Thus, it is important to devise an efficient and accurate method to identify members of the Hyrcanus Group [15], which is a prerequisite for malaria vector surveillance in practice [16, 17]. However, it is difficult to distinguish cryptic species in the Hyrcanus Group by morphological features [5, 18] because there of 1) the scarcity of trained morphologists in the field [19] and 2) the loss of taxonomic traits during daily surveillance activities, such as wing scales [15, 20].

Combined with morphological characteristics and molecular evidence [19, 21–23], the accuracy of mosquito identification has recently improved, both in fieldwork and scientific research. This was caused by 1) the rapid growth of molecular marker sequences in the GenBank database (http://www.ncbi.nlm.nih.gov/genbank/), 2) the consistency between barcoding results and traditional morphology-based taxonomy [24] and 3) the ability to extract enough deoxyribonucleic acid (DNA) for species identification by molecular methods from any life stage of individual mosquito [15, 25]. The gene region being used as the standard barcode for almost all animal species is an average 650 base-pair region in the mitochondrial cytochrome *c* oxidase 1 gene [25–28].

Compared with ribosomal DNA (rDNA), mitochondrial DNA (mtDNA) *COI* is advantageous because 1) its evolutionary rate is 5–10 times faster than that of rDNA [29], making it especially suitable for classification at the species level [28, 30]; 2) mtDNA is haplotype, and thus, there is no cloning step before sequencing, which is usually required when using rDNA as a molecular marker [31]; and 3) it has multiple copies, which makes amplification much easier [26]. The differences between *COI* sequences increase in higher taxonomic categories [32]. The *COI* barcoding gap is usually 2% within species [28]. High divergence of intraspecific distance is probably caused by recent geographic isolation, suggesting the presence of cryptic species [28, 33]. Thus, more complete sampling of the geographic range, greater distance among sample locations, and more diversified the sampled individuals, the more accurate the genetic divergences within and among species [19, 27, 30, 34]. As such, it will be easier to distinguish cryptic species from studies based on large geographic range, especially cryptic species with short historical divergence [27, 35, 36]. The distribution of mosquitoes is based on the geographic distribution of animal species, that is zoogeography [37]. However, in previous research on molecular phylogenetic reconstruction for mosquitoes, most studies [21, 33, 38–41] have targeted local or regional species from a small administrative area. Thus, it is probable that most studies did not comprehensively sample different species because the barcoding gap is correlated with the geographic scale of sampling [34, 42] and the sample size of target species [43, 44]. The exponential growth of GenBank *COI* sequences, accumulated from around the world [45, 46] makes it possible to study DNA barcoding at a more comprehensive scale for target species.

To further clarify the relationships among the *Anopheles hyrcanus* group species, and stablize the group, we combined our newly collected *COI* sequences and other sequences deposited in GenBank across a worldwide geographic range and applied different phylogenetic analytical methods to address the molecular phylogeny of the Hyrcanus Group. This research will provide a valuable tool for large-scale vector identification in practice and the planning of the malaria surveillance program in practice.

## Methods

### DNA extraction

A total of 33 dry museum specimens from the Hyrcanus Group, belonging to five species, *An. hyrcanus* (Pallas, 1771), *An. peditaeniatus* (Leicester, 1908), *An. sinensis*, *An. pullus* Yamada, 1937, and *An. liangshanensis* Kang, Tan, Cao, Cheng, Yang & Huang, 1984, were randomly chosen for DNA extraction. All of them were collected less than 7 years. Species identification was accomplished with the national key [9]. Collection localities and other specimen information are available in Table 1. One leg was removed from each adult specimen, transferred to a dry Eppendorf tube, and ground to powder. Then it was incubated in lysis buffer overnight at 56 °C. Additional steps followed the manufacturer’s instructions for the Qiagen® DNA blood & tissue kit. Voucher specimens were stored in the Herbarium of National Institute of Parasitic Diseases, Chinese Center for Disease Control and Prevention.

**Table 1:** List of *COI* sequences of the Hyrcanus Group obtained from this study

Species	Geographic localities	Genbank accession No.
*An. hyrcanus*	Xinjiang Prov., China	KT966851
	Xinjiang Prov., China	KT966852
	Xinjiang Prov., China	KT966853
	Xinjiang Prov., China	KU743222
	Xinjiang Prov., China	KU743223
	Xinjiang Prov., China	KU743224
	Xinjiang Prov., China	KU743225
	Xinjiang Prov., China	KU743226
	Xinjiang Prov., China	KU743227
*An. peditaeniatus*	Yunnan Prov., China	KT966854
	Yunnan Prov., China	KT966855
	Yunnan Prov., China	KT966856
	Yunnan Prov., China	KT966857
*An. sinensis*	Yunnan Prov., China	KT966858
	Yunnan Prov., China	KT966859
	Yunnan Prov., China	KT966860
	Yunnan Prov., China	KT966861
	Yunnan Prov., China	KT966862
	Yunnan Prov., China	KT966863
	Yunnan Prov., China	KT966864
	Yunnan Prov., China	KT966865
	Yunnan Prov., China	KT966866
	Yunnan Prov., China	KT966867
	Yunnan Prov., China	KT966868
	Yunnan Prov., China	KT966869
*An. pullus*	Liaoning Prov., China	KT966870
	Liaoning Prov., China	KT966871
	Liaoning Prov., China	KT966872
	Liaoning Prov., China	KT966873
	Liaoning Prov., China	KT966874
*An. liangshanensis*	Yunnan Prov., China	KU743228
	Yunnan Prov., China	KU743229
	Yunnan Prov., China	KU743230

### Sequence generation

Amplification of the *COI* region was performed with a universal primer pair. Universal primers LCO1490 (5′-GGT CAA CAA ATC ATA AAG ATA TTG G-3′, forward) and HCO2198 (5′-TAA ACT TCA GGG TGA CCA AAA AAT CA-3′) were used to amplify the *COI* sequences [47]. The amplified length was approximately 650 bp. The 25 μL reaction mixture contained 12.5 μL 2XTaq polymerase chain reaction (PCR) Master Mix (with dyes, DBI® Bioscience), 4 μL extracted DNA, and 6.5 μL ddH_2_O. The thermocycling profile consisted of one cycle of 2 min at 94 °C, five cycles of 30 s at 94 °C, 40 s at 45 °C, and 1 min at 72 °C, followed by 35 cycles of 30 s at 94 °C, 40 s at 51 °C, and 1 min at 72 °C, with a final extension at 72 °C (7 min). The PCR products were visualized on 1.2% 0.5XTBE agarose gels, then cleaned and sequenced by Shanghai Sangon (Shanghai, China).

### Search for *COI* sequences of the Hyrcanus Group in GenBank

Based on the index of Harbach [4], there are 25 species in this group. We searched and downloaded *COI* sequences for the Hyrcanus Group members deposited in GenBank (Additional file 2) with the keywords “(species name) & *COI*.” We checked and trimmed odd sequences with the highest 5% intraspecific distances or the lowest 5% interspecific distances [42, 48, 49] to avoid sequences posted in GenBank that contained errors [50–52]. Although the interspecific distances of *An. hyrcanus* and *An. pseudopictus* Grassi, 1899; *An. lesteri* and *An. paraliae* Sandosham, 1959; *An. sinensis*, *An. kleini* Rueda, 2005 and *An. belenrae* Rueda, 2005 were less than 5%, the independent sequences for those species were used in present study because the taxonomic validity of *An. pseudopictus*, *An. hyrcanus* sp_IR_, *An. paraliae*, and *An. kleini* are still controversial [5, 53, 54]. The information on locations for sample sequences and authors were also recorded (Additional file 2).

### Tree building

The *COI* sequence dataset was combined with our original fragments and records retrieved from GenBank. ClustalW2 [55] was used to align sequences using the default settings, and we created a neighbour joining (NJ) tree with 1 000 bootstraps. Based on Akaike Information Criterion (AIC), the best-fit model for the alignment was determined using Modeltest 3.7 [56], in cooperation with PAUP*4.0b10 [57]. Consequently, the construction of the maximum likelihood (ML) and Bayesian likelihood completed under the TVM + I + G model. *An. lindesayi* Giles, 1900 and *An. claviger* (Meigen, 1804) were used as outgroup taxa based on a previous study [33]. The ML tree was performed by RAxML-HPC2 v7.4.4 [58, 59] on the CIPRES portal (www.phylo.org/) [60] with 1 000 bootstraps. The Bayesian tree was built with MrBayes v3.2.1 [61], run for 1 million generations, with the first 25% generations discarded as burn-in. The trees were visualized and edited in FigTree v1.4.2 [62].

### Genetic diversity analysis

Pairwise distances within and between species were calculated using Kimura’s 2-parameter (K2P) distance model [63] in MEGA v5.10 [64]. DnaSP 5.10 [65] was applied to calculate the nucleotide diversity of *COI* sequences of each species, and we performed the neutrality tests for Fu’s *Fs* [66] and Tajima’s *D* value [67].

## Results

There were 463 *COI* sequences of the Hyrcanus Group in GenBank belonging to 18 species. There were no *COI* records for *An. chodukini* Martini, 1929, *An. engarensis* Kanda & Oguma, 1978, *An. hailarensis* Xu & Luo, 1998, *An. heiheensis* Ma, 1981, *An. hyrcanus* sp_IR_, *An. sineroides* Yamada, 1924, *An. vietnamensis* Nguyen, Tran & Nguyen, 1993, and *An. pseudosinensis* Baisas, 1935 in GenBank. The accession numbers of *COI* sequences, which we downloaded from GenBank, are available in Additional file 2, including the collection localities and author information. Because sequences submitted to GenBank came from labs worldwide, without further confirmation, we found some fragments that were distant from others in the same species (Additional file 2), but closer to sequences from their sister species, with peculiar phylogenetic positions in the pre-building phylogenetic tree (see Additional file 3). Thus, we removed them from further analyses.

Thirty-three newly collected sequences for five species (*An. hyrcanus*, *An. peditaeniatus*, *An. sinensis*, *An. pullus*, and *An. liangshanensis*) were included in this study. Amino acid translation showed that they were free of stop codons, indicating that none of them was rDNA sequences originating from mtDNA sequences. The GenBank accession numbers are listed in Table 1.

**Fig. 1:**
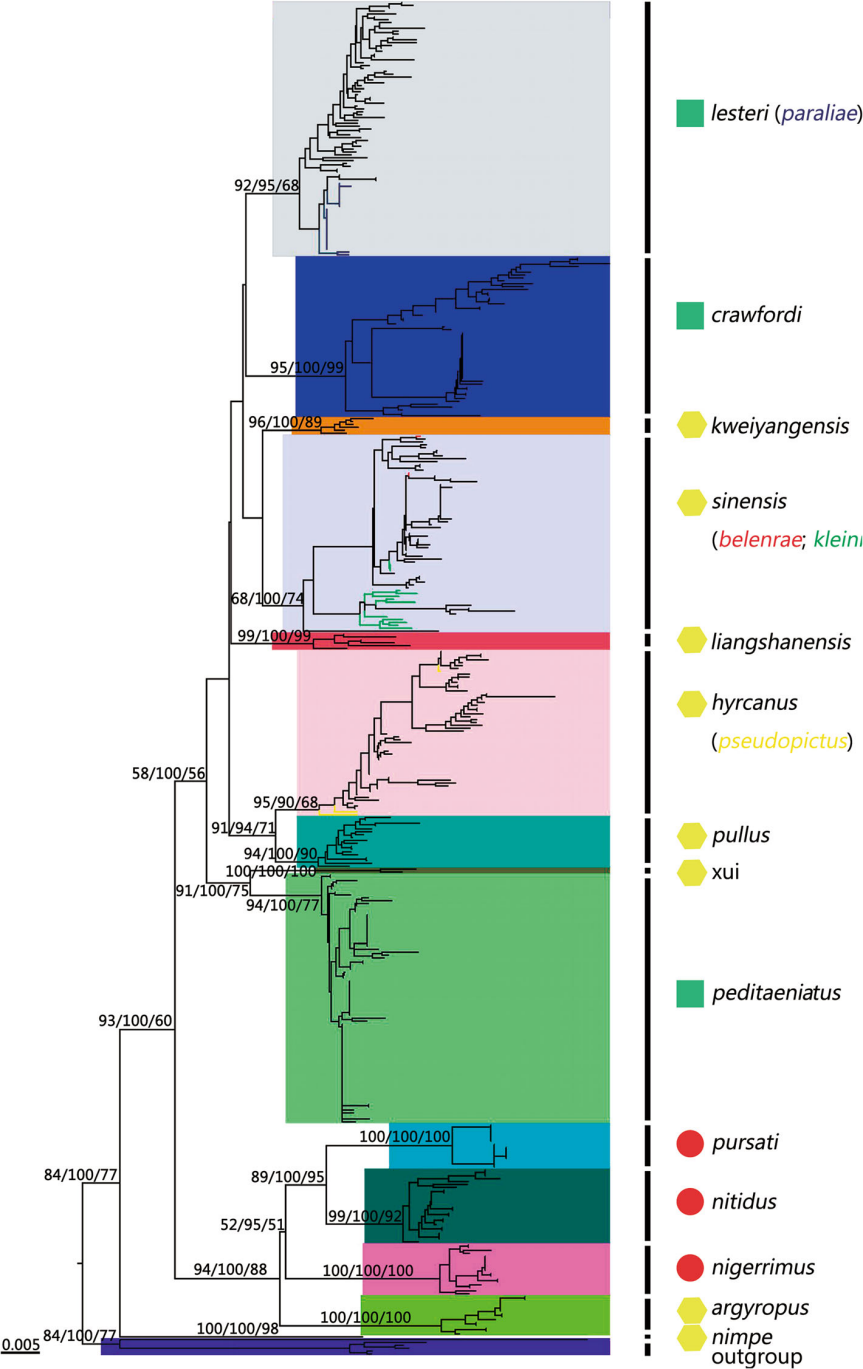
Neighbour joining tree based on *COI* sequences deposited in GenBank and our original data for the Hyrcanus Group. Bootstrap values (1 000 replicates, not shown for less than 50%) of Neighbour Joining, Bayesian, and Maximum likelihood analyses are shown above the main lineages, respectively. Lineage designation is indicated on the right. Branches representing *COI* sequences of *An. belenrae*, *An. kleini*, *An. pseudopictus*, and *An. paraliae* are indicated by *red*, *green*, *yellow*, and *blue*, respectively. The geometric shapes correspond to the different subgroups of the Hyrcanus Group, according to the classification of Harbach [4]. (*green square*) Lesteri Subgroup; (*red circle*) Nigerrimus Subgroup; (*yellow hexagon*) unclassified species. Bars represent 0.1 substitutions per site. *An. lindesayi* and *An. claviger* were used as outgroup taxa

**Fig. 2:**
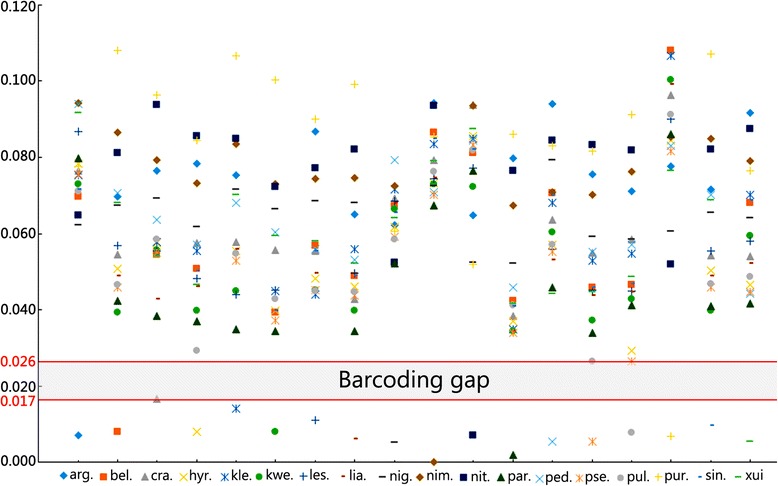
Plot of K2P distance of the 18 Hyrcanus Group members determined using NJ-K2P distances. Y-axis: genetic divergence; X-axis: Hyrcanus group members. arg. = *An. argyropus*; bel. = *An. belenrae*; cra. = *An. crawfordi*; hyr. = *An. hyrcanus*; kle. = *An. kleini*; kwe. = *An. kweiyangensis*; les. = *An. lesteri*; lia. = *An. liangshanensis*; nig. = *An. nigerrimus*; nim. = *An. nimpe*; nit. = *An. nitidus*; par. = *An. paraliae*; ped. = *An. peditaeniatus*; pul. = *An. pullus*; pur. = *An. pursati*; sin. = *An. sinensis*; xui = *An. xui*

After combining *COI* records from GenBank with our original sequences and excluding suspicious fragments, 466 sequences of 18 Hyrcanus Group members were used for analyses of genetic diversity indices and reconstruct phylogenetic trees. The topology of the NJ tree, ML tree, and Bayesian tree were almost identical for the major lineages, although node confidence values were slightly different among the three (Fig. 1). Hence, only the NJ tree is presented here. The tree showed that the group could be divided into at least three main clusters. The first cluster was solely composed of *An. nimpe* Nguyen, Tran & Nguyen, 2000, which was coincidence with genetic distance analysis. *An. nimpe* exhibited extensive interspecific divergences (the minimum distance was 0.067) with other Hyrcanus Group members (Table 2, Fig. 2). The second cluster consisted of *An. nigerrimus* Giles, 1900, *An. nitidus* Harrison, Scanlon & Reid, 1973, *An. pursati* Laveran, 1902, and *An. argyropus* (Swellengrebel, 1914), and the third cluster included *An. sinensis*, and *An. belenrae*, *An. kleini* grouped; *An. lesteri and An. paraliae* grouped; *An. crawfordi* Reid, 1953; *An. hyrcanus* and *An. pseudopictus* grouped; *An. liangshanensis*; *An. kweiyangensis* Yao & Wu, 1944; *An. peditaeniatus*; *An. sineroides*; and *An. pullus* Yamada, 1937. Almost all node-linking sequences of individuals of the same species had a high bootstrap value. However, the relationships between *hyrcanus*/*pseudopictus*, *lesteri*/*paraliae*, and *sinensis*/*belenrae*/*kleini* were unclear. They exhibited very low pair-wise distance values (Table 2), and formed monoclades with high node confidence values (Fig. 1).

**Table 2:** Mean intra- and interspecific K2P distances of the *COI* gene in 18 Hyrcanus Group members

Species	n	arg.	bel.	cra.	hyr.	kle.	kwe.	les.	lia.	nig.	nim.	nit.	par.	ped.	pse.	pul.	pur.	sin.	xui
arg.	14	**0.007**																	
bel.	2	0.070	**0.008**																
cra.	56	0.077	0.055	**0.017**															
hyr.	55	0.078	0.051	0.058	**0.008**														
kle.	13	0.075	0.024*	0.058	0.055	**0.014**													
kwe.	6	0.073	0.039	0.056	0.040	0.045	**0.008**												
les.	63	0.087	0.057	0.055	0.048	0.044	0.045	**0.011**											
lia.	6	0.065	0.049	0.043	0.046	0.056	0.040	0.050	**0.006**										
nig.	18	0.062	0.067	0.069	0.062	0.072	0.066	0.068	0.068	**0.005**									
nim.	1	0.094	0.086	0.079	0.073	0.083	0.073	0.074	0.075	0.073	n/c								
nit.	26	0.065	0.081	0.094	0.086	0.085	0.072	0.077	0.082	0.052	0.094	**0.007**							
par.	26	0.080	0.042	0.038	0.037	0.035	0.035	0.020*	0.035	0.052	0.067	0.077	**0.002**						
ped.	87	0.094	0.071	0.064	0.057	0.068	0.060	0.056	0.053	0.079	0.071	0.084	0.046	**0.005**					
pse.	3	0.076	0.046	0.055	0.007*	0.053	0.037	0.045	0.044	0.059	0.070	0.083	0.034	0.055	**0.005**				
pul.	18	0.071	0.047	0.058	0.029	0.055	0.043	0.045	0.045	0.058	0.076	0.082	0.041	0.057	0.026	**0.008**			
pur.	16	0.078	0.108	0.096	0.084	0.107	0.100	0.090	0.099	0.060	0.085	0.052	0.086	0.083	0.082	0.091	**0.007**		
sin.	54	0.072	0.009*	0.054	0.050	0.023*	0.040	0.055	0.049	0.065	0.085	0.082	0.041	0.070	0.046	0.047	0.107	**0.010**	
xui	2	0.092	0.068	0.054	0.047	0.070	0.059	0.058	0.052	0.064	0.079	0.087	0.042	0.044	0.045	0.049	0.077	0.069	**0.005**

The numbers of intraspecific distances are shown in boldface for clarity. Numbers underlined indicate the highest intraspecific distance and the lowest interspecific distance. *n* = No. of sequences. The interspecific distances of *hyrcanus*/*pseudopictus*, *lesteri*/*paraliae*, *sinensis*/*belenrae*/*kleini*, were highlighted with an asterisk. arg. = *An. argyropus*; bel. = *An. belenrae*; cra. = *An. crawfordi*; hyr. = *An. hyrcanus*; kle. = *An. kleini*; kwe. = *An. kweiyangensis*; les. = *An. lesteri*; lia. = *An. liangshanensis*; nig. = *An. nigerrimus*; nim. = *An. nimpe*; nit. = *An. nitidus*; par. = *An. paraliae*; ped. = *An. peditaeniatus*; pul. = *An. pullus*; pur. = *An. pursati*; sin. = *An. sinensis*

Excluding these polytypic species above, based on the mtDNA *COI* sequence, the average K2P distances between and within the Hyrcanus Group species were 0.064 (range 0.027–0.108) and 0.008 (range 0.002–0.017), respectively (Table 2). On average, the differences between congroup species were 8-fold higher than the differences within species. The maximum K2P distance within species was in *An. crawfordi* (0.017), and the minimum K2P distance between the Hyrcanus Group members was 0.026 (Fig. 2).

It should be noted that *An. belenrae* and *An. kleini* had a genetic distance of 0.024 from each other, and 0.009 and 0.023, respectively, with *An. sinensis*. In addition, the distance between *hyrcanus* and *pseudopictus* was 0.007, and it was 0.020 between *lesteri* and *paraliae*.

The genetic diversity indices and the results of neutrality tests are showed in Table 3. Both Fu’s *Fs* and Tajima’s *D* values were significant in *An. lesteri* and *An. pullus*, suggesting past population expansion.

**Table 3:** Genetic diversity indices and neutrality tests of the *COI* gene in 14 Hyrcanus Group members

species	n	S	Pi	h	Hd	Fu’s *Fs*	Tajima’s *D*
*An. argyropus*	14	13	0.00576	7	0.89	−0.269	−0.32403
*An. crawfordi*	56	37	0.01910	31	0.902	−10.400**	0.09433
*An. hyrcanus*	55	27	0.00880	25	0.925	−13.586**	−1.25768
*An. kleini*	13	20	0.01230	11	0.962	−3.224*	0.35139
*An. kweiyangensis*	6	8	0.0054	6	1	−3.07918*	−0.39875
*An. lesteri*	63	58	0.01026	57	0.997	−74.707**	−1.74894*
*An. liangshanensis*	6	10	0.00628	6	1.000	−2.734	−0.79480
*An. nigerrimus*	18	16	0.00631	10	0.882	−1.923	−0.61570
*An. nitidus*	26	24	0.00816	14	0.948	−2.844*	−0.53529
*An. paraliae*	26	5	0.00150	5	0.591	−0.832	−0.70434
*An. peditaeniatus*	87	16	0.00533	19	0.837	−9.149**	−1.08597
*An. pullus*	18	23	0.00707	16	0.987	−11.407**	−1.70855*
*An. pursati*	16	7	0.00540	3	0.692	5.031	2.40596
*An. sinensis*	54	29	0.00906	28	0.911	−18.570**	−1.44523

*n* = number of sequences; *S* = number of polymorphic sites; *pi* = nucleotide diversity; *h* = number of haplotypes; *Hd* = haplotype diversity. The significance of Fu’s *Fs* or Tajima’s *D* values is indicated by asterisks (* *P* < 0.05; ** *P* < 0.01). Species represented by <3 specimens were excluded from the analyses

## Discussion

The suspicious records for *COI* found in this study, as evidenced by their pairwise distances and phylogenetic positions, may have been caused by misidentification of specimens in previous studies. Misidentified *COI* sequences were detected in mosquitoes in a previous study [52]; however this was possibly caused by the presence of other cryptic species. Although it is possible that they were morphologically misidentified or cryptic species, we omitted them from the analyses of genetic diversity indices and tree building. The maximum intra-specific distance of Hyrcanus Group was 0.017. A same value was obtained for *Anopheles* by other labs [33, 38].

The Hyrcanus Group was monophyletic, as described by other authors [4, 68]. However, the subgroup division using *COI* was not the same as those based on morphologic characteristics [69, 70], nor as results obtained from nuclear marker phylogeny [5, 54, 68, 71]. The topology of *COI* tree obtained from this study was similar with the results described by Wijit et al. [72], with the same molecular marker, *COI*. Although the applied *COI* sequences in the former study did not contain sequences for *An. nimpe*, the NJ tree showed the other two main clusters. In the one comprised of *An. nigerrimus*, *An. nitidus*, *An. pursati*, and *An. argyropus*, the former three species were grouped into the Nigerrimus Subgroup. The remaining studied species were placed in the other cluster, including *sinensis* (unassociated species) and the Lesteri Subgroup (*crawfordi*, *lesteri*, *paraliae*, *peditaeniatus*). The trees from both studies indicated that the Lesteri Subgroup, as classified by morphological features, was not monophyletic.

Because of *ITS2* sequence differences and the discrepancy of morphological identification, Rueda [16] distinguished and named two new species, *An. belenrae* and *An. kleini*, from *An. sinensis*. However, the pairwise differences of *COI* among the three species were below the lower threshold of the barcoding gap (Table 2, Fig. 2). In the phylogenetic tree (Fig. 1), the three species formed a monoclade with a high node confidence (NJ 68%; Bayes 100%; ML 74%). This suggested that the gene introgression at the mtDNA likely happened during species expansion [27, 73]. The hybridization experiments under laboratory conditions supported natural hybridization between *An. sinensis* and *An. kleini* [74]. It was inferred that the same situation probably happen between *belenrae* and *sinensis* in field.

*Anopheles lesteri* from Korea and *An. paraliae* from Thailand were suggested as conspecifics by Taai et al. [75], inferred from crossing experiments and molecular analyses. It is in agreement with the current study. The pairwise distance between *An. lesteri* and *An. paraliae* was 0.019, and the two species could not be distinguished in the phylogenetic tree (Fig. 1). *An. lesteri* is widely distributed across the Palaearctic and Oriental regions, north into the Primorsk region and Russia, and south to Philippines, Malaysia [9]. *An. anthropophagus* is endemic to China as evidenced by a synonym of *An. lesteri* [76, 77]. All *COI* sequences of *An. paraliae* in GenBank were obtained from specimens that were collected from Thailand. It is likely that *An. lesteri*, *An. anthropophagus*, and *An. paraliae* belong to a single species. The morphological differences among the three species were likely a result of recent geographic isolation.

The *COI* sequences of *An. hyrcanus* and *An. pseudopictus* were almost identical, with a distance of 0.008. *An. pseudopictus* clustered within the *An. hyrcanus* lineage in *COI* tree. Poncon et al. [53] demonstrated that the two species and their intermediate form were indistinguishable by nuclear markers. However, there was no further study on crossing experiments to support this supposition.

Two *COI* fragments of *An. crawfordi* (KF830735.1; KF830736.1) in GenBank collected from China (direct submission) were clustered with the lineage of *An. xui* Dong, Zhou, Dong & Mao, 2007 in the phylogenetic tree (Additional file 3). They were closer to sequences of *An. xui* than to those of their conspecifics. It implied that *An. crawfordi* might not exist in China, or quite possibly that the specimens were misidentified.

Because genes submitted to GenBank were without confirmation, there was probably some error sequences in the database [50, 78]. In this study, some sequences excluded in later phylogenetic analyses were related to authors (see Additional file 2) who submitted them to GenBank. Almost all *COI* sequences of the Hyrcanus Group submitted by some authors had peculiar phylogenetic positions; some even had considerable distances from other sequences from the same species. The validity of those data needs further research. It is possible that they could have been cryptic taxa.

## Conclusions

The large data analysis showed that the *COI* barcoding gap (K2P distance) of the Hyrcanus Group species was 0.017 to 0.026. The average conspecific K2P divergence was 0.008 (range 0.002–0.017), whereas sequence divergence between congroup species averaged at 0.064 (range 0.026–0.108). The *COI* tree showed that the group could be divided into at least three main clusters. The first cluster contained *An. nimpe*; the second was composed of the Nigerrimus Subgroup and *An. argyropus*; the third cluster was comprised of the Lesteri Subgroup and other unassociated species. It was consistent with former phylogenetic analyses of the Hyrcanus Group with the same gene based on small sample sizes [72], but contradicted the morphological and rDNA *ITS2*-based classification when sorting out subgroups. In addition, phylogenetic analysis suggested that ancient hybridizations probably happen among the three species, *An. sinensis*, *An. belenrae*, and *An. kleini*. It supported that *An. paraliae* was synonymized with *An. lesteri*, whereas *An. pseudopictus* and *An. hyrcanus* may belong to a single species, as evidenced from extremely low interspecific genetic divergence (0.020 and 0.007, respectively), and their phylogenetic positions.

The neutrality tests indicated that several Hyrcanus Group members, *An. lesteri*, *An. sinensis*, *An. hyrcanus*, *An. pullus*, *An. peditaeniatus*, and *An. pseudopictus*, might have experienced population expansion or genetic hitchhiking. Almost all of these species are widespread and some of them have the capacity of malaria transmission.

*An. sinensis*, *An. kleini* and *An. belenrae* have been proved as suspected malaria vectors in South Korea [20, 79–81]. It can be inferred that closely related species may possess similar susceptibility to plasmodium infection. In addition, it has the possibility of natural hybridization happening among closely related species [27]. Therefore, we need to pay attention to *An. paraliae*, *An. pseudopictus* in future surveillance as well, since that their sister species has been incriminated as vectors of malaria [9, 82]. Integrated molecular phylogeny research combining both mtDNA and rDNA for the *Anopheles hyrcanus* group is underway in our lab.

## Additional files

Additional file 1: Multilingual abstracts in the five official working languages of the United Nations. (PDF 818 kb)

Additional file 2: List of *Anopheles hyrcanus* group *COI* sequences deposited in GenBank and obtained from this study, with GenBank accession numbers, geographic localities, and corresponding authors. Suspicious sequences with high intraspecific genetic distance, and peculiar phylogenetic positions were annotated. (XLSX 33 kb)

Additional file 3: Neighbour-joining phylogenetic tree of *COI* in the Hyrcanus Group showing suspicious sequences. Bootstrap values are shown above the main lineages. Lineage designation is indicated on the right. The geometric shape (red square) corresponds to suspicious sequences of the Hyrcanus Group in GenBank. (PDF 617 kb)

## Abbreviations

AIC: Akaike information criterion
COI: Cytochrome c oxidase subunit I
DNA: Deoxyribonucleic acid
K2P distance: Kimura 2-parameter distance
ML: Maximum likelihood
mtDNA: Mitochondrial DNA
NJ: Neighbour joining
PCR: Polymerase chain reaction
rDNA: Ribosomal DNA
TBE: Tris/Borate/EDTA
