# Supplementary material for: Molecular phylogeny of Anopheles hyrcanus group (Diptera: Culicidae) based on mtDNA COI
Source: Infect Dis Poverty. 2017 May 8;6:61. doi: 10.1186/s40249-017-0273-7 (PMC5421329; doi:10.1186/s40249-017-0273-7)
Supplement: Supplementary file 1 — Multilingual abstracts in the five official working languages of the United Nations. (PDF 818 kb) [file 40249_2017_273_MOESM1_ESM.pdf]

Translation of the abstract into the five official working languages of the United Nations

تطور السُّلالات الجزيئي من مجموعة الأنوفيليس الهيركانية (ذوات الجناحين: البعوضيات) على أساس أوكسيداز السيتوكروم للحمض النووي الميتوكوندري

يوان فانج، ون تشى شي وتشانج يي

ملخص

خلفية: المجموعة الأنوفيليس الهيركانية، التي تضم 25 نوعا على الأقل، وتتوزع على نطاق واسع في المناطق الشرقية والمنطقة القطبية القديمة، اعتُبر بعض أعضاء المجموعة النواقل المسببة للملاريا وغيرها من الأمراض التي تنتقل عن طريق البعوض. ومن الصعب تحديد أعضاء مجموعة هيركانوس عن طريق المميزات الشكلية. وهكذا، فقد اقترح تطور السُّلالات الجزيئي كأسلوب تكميلي هام للتصنيف المورفولوجي التقليدي.

الطريقة: استنادا إلى قاعدة بيانات بنك الجينات وبيانات الدراسة الأصلية، استخدمنا 466 تسلسل أوكسيداز سيتوكروم مُتَقَدِّرِيّ للحمض النووي التي تنتمي إلى 18 نوعا لإعادة بناء تطور السُّلالات الجزيئي من المجموعة هيركانوس عبر نطاقها الجغرافي في جميع أنحاء العالم.

النتائج: كانت النتائج على النحو التالي: 1) كان متوسط انحراف K2P المناوع 0.008 (المدى 0.002-0.017)، في حين أن انحراف التسلسل بين أنواع المجموعة المشتركة في المتوسط 0.064 (المدى 0.026-0.108). 2) كانت طوبولوجيا شجرة تسلسل أوكسيداز السيتوكروم لمجموعة هيركانوس تتفق عموما مع التصنيف المورفولوجي الكلاسيكي من حيث تصنيف الأنواع، ولكن اختلفت في القسم الفرعي. في شجرة تسلسل أوكسيداز السيتوكروم، تم تقسيم المجموعة إلى ثلاث مجموعات رئيسية على الأقل. المجموعة الأولى Anopheles nimpe، وتألفت الثانية من المجموعة الفرعية Nigerrimus وAnopheles argyropus، وتتألف المجموعة الثالثة من المجموعة الفرعية Lesteri والأنواع غير المصاحبة الأخرى. 3) أشار تحليل النشوء وتطور تسلسل أوكسيداز السيتوكروم أن تهجينا قديما ربما وقع بين الأنواع الثلاثة المرتبطة ببعضها ارتباطا وثيقا وهي Anopheles sinensis، Anopheles belenrae، Anopheles kleini، 4) أيدت النتائج أن Anopheles paraliae هي مضارع محتمل لـ Anopheles lesteri، ومن الممكن أن تكون Anopheles pseudopictus والأَنُوفيلةُ الهيركانِيَّة هما نفس النوع، كما هو واضح من الاختلاف الجيني المنخفض للغاية بين الأنواع (0.020 و0.007 على التوالي) ووضعهم في النشوء والتطور.

الاستنتاجات: باختصار، أعدنا بناء تطور السُّلالات الجزيئي وتحليل التباين الوراثي للمجموعة هيركانوس باستخدام تسلسل أوكسيداز السيتوكروم المُتَقَدِّرِيّ. وتشير النتائج التي توصلنا إليها أنه في المستقبل عند مراقبة الملاريا، ينبغي أن نولي ليس فقط الكثير من الاهتمام لتلك النواقل المعروفة من الملاريا، ولكن أيضا الكثير من الأنواع ذات الصلة الوثيقة.

Translated from English version into Arabic by Mahmoud Sami, through

TRANSLATORS
WITHOUT BORDERS

基于线粒体 *COI* 序列的赫坎按蚊种团分子系统进化研究

Yuan Fang, Wen-Qi Shi and Yi Zhang

摘要：

引言：目前上已报道的赫坎按蚊种团，具有有效学名的为 25 种，广泛分布于古北界和东洋界。其中一些蚊种被确定为是传播疟疾和其他媒传疾病的重要媒介。然而，从形态学角度很难区分赫坎按蚊种团蚊种。而分子生物学可作为传统形态分类的佐证，为阐明种团内各蚊种

的亲缘关系提供重要依据。
方法：本文将基于全球地理生态分布的视野，结合 GenBank 和本实验室所获的分子片段，利用分属 18 个种的 466 条 *COI* 序列，重建赫坎按蚊种团蚊种分子系统进化关系。
结果：结果显示：1）赫坎按蚊种团蚊种平均种内遗传距离为 0.008（0.002-0.017），平均种间遗传距离为 0.064（0.026-0.108）。2）在蚊种界定上，*COI* 进化树的拓扑结构与形态分类结果基本一致，但在亚种群的划分上存在分歧。从 *COI* 树上看出，该种团至少可划分为三个主要分枝。第一分枝目前仅有尼姆按蚊；第二个分枝包含形态分类界定的最黑按蚊亚种团和银足按蚊；第三分枝由雷氏按蚊亚种团和其余形态分类未作划分的蚊种组成。3）COI 基因分子系统进化显示中华按蚊、比伦按蚊和克莱按蚊，三者之间很可能曾存在自然杂交。4）结合遗传距离和进化树拓扑结构分析，巴拉按蚊很可能是雷氏按蚊的同物异名，伪色按蚊和赫坎按蚊存在为同一物种的可能。
结论：综上所述，利用线粒体基因 *COI* 序列，我们重建了赫坎按蚊种团分子系统进化，并进行了遗传距离分析，对现场工作具有一定的指导意义。在今后的疟疾防控中，我们不仅要监控已知的传疟媒介，同时也要关注它们的近缘种。

Translated from English version into Chinese by Yuan Fang

Phylogénie moléculaire du groupe *Anopheles hyrcanus* (diptères : Culicidae) sur la base de la *COI* de l’ADN mitochondriale

Yuan Fang, Wen-Qi Shi et Yi Zhang

Résumé
Contexte : le groupe *Anopheles hyrcanus* qui comprend au moins 25 espèces est largement réparti dans les régions orientale et paléarctique. Certains membres du groupe sont supposés être des vecteurs du paludisme et d’autres maladies transmises par les moustiques. Il est difficile d’identifier les membres du groupe Hyrcanus à l’aide de caractéristiques morphologiques. La phylogénie moléculaire a donc été proposée à titre d’importante méthode complémentaire à la taxonomie morphologique traditionnelle.
Méthodes : sur la base de la base de données GenBank et de nos données d’étude originales, nous avons utilisé 466 séquences de *COI* d’ADN mitochondriale appartenant à 18 espèces afin de reconstruire la phylogénie moléculaire du groupe Hyrcanus parmi son étendue géographique mondiale.
Résultats : les résultats sont comme suit : 1) La divergence moyenne K2P conspécifique s’élevait à 0,008 (intervalle : 0,002 – 0,017), tandis que la divergence séquentielle moyenne entre des espèces d’un même groupe atteignait 0,064 (intervalle : 0,026 – 0,108). 2) La topologie de l’arbre *COI* du groupe Hyrcanus correspondait généralement à une taxonomie morphologique classique en termes de classification des espèces mais divergeait en termes de division par sous-groupe. Dans l’arbre *COI*, le groupe était divisé en au moins trois principaux ensembles. Le premier ensemble contenait *An. Nimpe* ; le deuxième était composé du sous-groupe Nigerrimus et *An. Argyropus* et le troisième ensemble regroupait le sous-groupe Lesteri et d’autres espèces non associées. 3) L’analyse phylogénétique de la *COI* a indiqué la survenue possible d’anciennes hybridations parmi les trois espèces étroitement liées suivantes : *An. sinensis*, *An. belenrae* et *An. kleini*. 4) D’après les résultats,

An. paraliae constitue un synonyme probable du *An. Lesteri* et il est possible que le *An. pseudopictus* et le *An. hyrcanus* fassent partie de la même espèce, comme le prouve leur divergence génétique interspécifique extrêmement faible (respectivement 0,020 et 0,007) et leurs positions phylogénétiques.
Conclusions : pour résumer, nous avons reconstruit la phylogénie moléculaire et avons analysé la divergence génétique du groupe Hyrcanus à l'aide de séquences de la *COI* mitochondriale. Nos résultats suggèrent qu'il conviendrait non seulement de tenir compte de ces vecteurs connus du paludisme, mais aussi de leurs espèces étroitement liées dans le cadre des futurs programmes de surveillance du paludisme.

Translated from English version into French by eric ragu, through

Molecular phylogeny of *Anopheles hyrcanus* group (Diptera: Culicidae) based on mtDNA *COI*

Yuan Fang, Wen-Qi Shi and Yi Zhang

Реферат
Фон: Группа Малярийный Hyrcanus, которая включает по меньшей мере 25 видов, широко распространена в Восточном и регионов Палеарктики. Некоторые члены группы инкриминировали в качестве переносчиков малярий и других переносимых комарами заболеваний. Трудно определить членов Гиркана группы по морфологическим признакам. Таким образом, молекулярная филогения была предложена как важный дополнительный метод к традиционной морфологической классификации.
Методы: На основе данных Генбанка и наших оригинальных данных исследований, мы использовали 466 митохондриальных последовательностей DNA *COI*, принадлежащих к 18 видам реконструировать молекулярную филогению при группе Hyrcanus через свою всемирную географию.
Результаты: Итоги таковы, 1) Средняя дивергенции для K2P состовляет 0.008 (в диапазоне 0.002 – 0.017), тогда как расходимость последовательности между congroup виды усредненные 0.064 (в диапазоне 0.026 – 0.108). 2) Топология *COI* дерево Гирканской группы в целом соответствует классической морфологической таксономии с точки зрения классификации видов, но не соглашалась на разделении подгруппы. В *COI* дереве группа была разделена на, по крайней мере, три основные группы. Первый кластер содержит *An. nimpe*; вторая состояла из подгруппы Nigerrimus Subgroup и *An. argyropus*; и третий кластер состоял из подгруппы Lesteri и других несвязных пород. 3) Филогенетический анализ *COI* показали, что древние гибридизации, вероятно, произошли между тремя родственными видами, как *An. sinensis*, *An. belenrae*, и *An. kleini*. 4) Результаты поддержали *An. paraliae* как возможный синонимом *An. lesteri*, и это было возможно, что *An. pseudopictus* и *An. hyrcanus* были одинаковым видом, как видно из их чрезвычайно низкой межвидовой генетической

дивергенции (0.020 и 0,007, соответственно) и их филогенетического положения.
Заключение: В резюме, мы реконструировали молекулярную филогению и проанализировали генетические расхождения при группы Hyrcanus с использованием митохондриальной *COI* последовательности. Наши результаты позволяют предположить, что в будущем надзор за малярией, мы должны не только обращать внимание на известные переносчики малярии, а также их близкородственные виды.

Translated from English version into Russian by Hao-Qi Zhang

Filogenia molecular del grupo *Anopheles hyrcanus* (Díptero: Culicidae) basado en mtADN *COI*

Yuan Fang, Wen-Qi Shi y Yi Zhang

Sinopsis

Antecedentes: El grupo *Anopheles hyrcanus*, que incluye al menos 25 especies, tiene una amplia distribución en las regiones oriental y paleoártica. Algunos miembros de este grupo se han considerado vectores de malaria y de otras enfermedades transmitidas por mosquitos. Resulta difícil identificar a los miembros del grupo Hyrcanus por rasgos morfológicos, así que se ha propuesto la filogenia molecular como método importante que complemente a la taxonomía morfológica tradicional.
Métodos: Basándose en la base de datos GenBank y en los datos originales de nuestro estudio, empleamos 466 secuencias de ADN *COI* mitocondrial pertenecientes a 18 especies para reconstruir la filogenia molecular del grupo Hyrcanus en toda su área de distribución geográfica.
Resultados: Los resultados son los siguientes, 1) La divergencia K2P media entre coespecíficos es de 0,008 (rango de entre 0,002 – 0,017), mientras que la media de divergencia secuencial entre especies cogrupales es de 0,064 (rango de entre 0,026 – 0,108). 2) En general, la topología del árbol *COI* del grupo Hyrcanus es coherente con la taxonomía morfológica clásica en cuanto a clasificación de especies, pero no en cuanto a división de subgrupo. En el árbol *COI*, el grupo está dividido en al menos tres clústeres: en el primero se incluye *An nimpe*, el segundo está formado por el subgrupo Nigerrimus y *An. argyropus*, y el tercer clúster está formado por el subgrupo Lesteri y otras especies no asociadas. 3) El análisis filogenético del *COI* indica que probablemente se produjeron hibridaciones antiguamente entre las tres especies con una relación cercana, *An. sinensis*, *An. belenrae*, y *An. kleini*. 4) Los resultados apoyan la teoría de que *An. paraliae* sea un sinónimo de *An. lesteri*, y es posible que *An. pseudopictus* y *An. hyrcanus* sean la misma especie, tal y como indica su extremadamente baja divergencia genética interespecífica (0,020 y 0,007, respectivamente) y sus posiciones filogenéticas.
Conclusiones: En resumen, se reconstruyó la filogenia molecular y se analizó la divergencia genética del grupo Hycarnus mediante secuencias *COI* mitocondriales. Nuestros resultados indican que en un futuro el seguimiento de la malaria debería centrarse no sólo en los vectores conocidos, sino también en especies con una relación cercana.

Translated from English version into Spanish by Denis Smyth, through

TRANSLATORS
WITHOUT BORDERS
