# Supplementary material for: Molecular phylogeny of Anopheles hyrcanus group (Diptera: Culicidae) based on mtDNA COI
Source: Infect Dis Poverty. 2017 May 8;6:61. doi: 10.1186/s40249-017-0273-7 (PMC5421329; doi:10.1186/s40249-017-0273-7)
Supplement: Supplementary file 3 — Neighbour-joining phylogenetic tree of COI in the Hyrcanus Group showing suspicious sequences. Bootstrap values are shown above the main lineages. Lineage designation is indicated on the right. The geometric shape (red square) corresponds to suspicious sequences of the Hyrcanus Group in GenBank. (PDF 617 kb) [file 40249_2017_273_MOESM3_ESM.pdf]

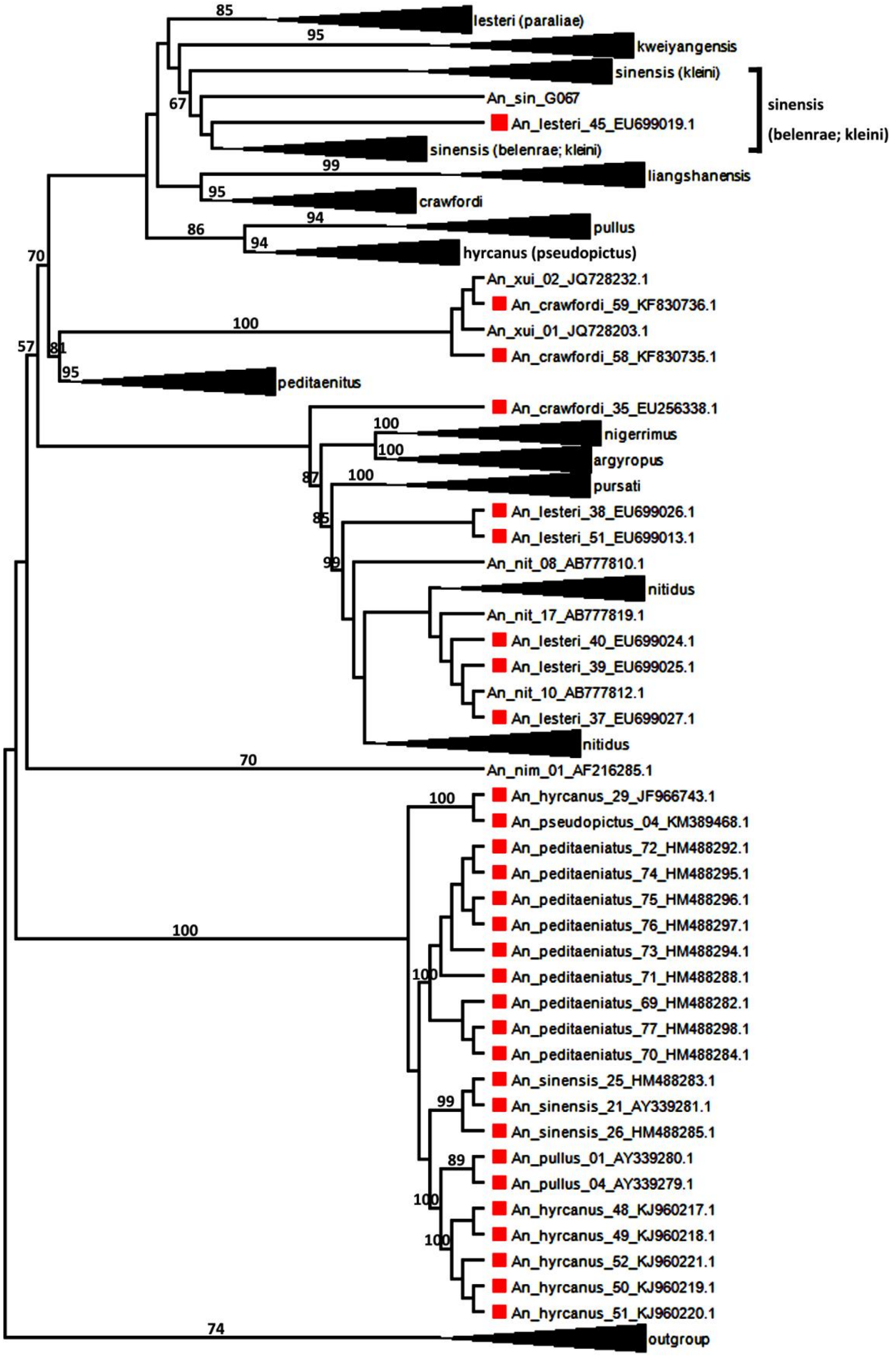

Additional file 2. **Neighbor-joining phylogenetic tree of *COI* in the Hyrcanus Group showing suspicious sequences.** Bootstrap values are shown above the main lineages. Lineage designation is indicated on the right. The geometric shape ■ corresponds to suspicious sequences of the

Hyrcanus Group in GenBank. The *An. lindesayi* and *An. claviger* were used as outgroup taxa.

cra=*crawfordi*; hyr= *hyrcanus*; ped=*peditaeniatus*; pul=*pullus*; sin=*sinensis*.
